# Supplementary material for: Relationship between Cerebrospinal Fluid Matrix Metalloproteinases Levels and Brain Amyloid Deposition in Mild Cognitive Impairment
Source: Biomolecules. 2021 Oct 11;11(10):1496. doi: 10.3390/biom11101496 (PMC8533797; doi:10.3390/biom11101496)
Supplement: Supplementary file 1 [file biomolecules-11-01496-s001.zip › biomolecules-1360111-supplementary.pdf]

Supplementally table S1. Comparison of CSF MMP and TIMP levels between APOE4-negative and APOE4-positive groups

	APOE4-negative (n=22)	APOE4-positive (n=11)	*p*
MMP-2	7795.1 ± 2404.5	7997.1 ± 3525.7	0.955
MMP-7	54.2 ± 25.3	65.9 ± 34.4	0.440
MMP-12	34.7 ± 41.3	59.7 ± 107.2	0.380
TIMP-1	26872.9 ± 8218.7	29235.7 ± 8665	0.355
TIMP-2	23585.7 ± 6007.7	25415 ± 6498.2	0.778

MMP, matrix metalloproteinase; TIMP, tissue inhibitor of metalloproteinase. [1] A *p*-value < 0.05 was considered statistically significant.

Supplementally table S2. Comparison of CSF MMP and TIMP levels between individuals with and without vascular risk factors.

	Vascular risk factor (+) (n=27)	Vascular risk factor (-) (n=6)	*p*
MMP-2	8026.5 ± 2983.7	7124 ± 1452.8	0.733
MMP-7	56.3 ± 28.3	66.2 ± 31.6	0.508
MMP-12	47.4 ± 75.7	23.4 ± 26.8	0.545
TIMP-1	27971.1 ± 8881.2	26262.7 ± 5388.1	0.600
TIMP-2	24209.8 ± 6582.2	24131.3 ± 3956.7	0.982

MMP, matrix metalloproteinase; TIMP, tissue inhibitor of metalloproteinase. [1] A *p*-value < 0.05 was considered statistically significant.
